# The achievable resolution for X-ray imaging of cells and other soft biological material

**DOI:** 10.1107/S2052252520002262

**Published:** 2020-03-07

**Authors:** Colin Nave

**Affiliations:** a Diamond Light Source Ltd, Harwell Science and Innovation Campus, Didcot, Oxfordshire OX11 0DE, UK

**Keywords:** X-ray imaging, coherent diffraction imaging, absorption-based imaging, Compton scattering, biological cells, radiation damage, dose-fractionation theorem

## Abstract

X-ray imaging of soft materials is often difficult because of the low contrast of the components. This particularly applies to frozen hydrated biological cells where the feature of interest can have a similar density to the surroundings. As a consequence, a high dose is often required to achieve the desired resolution. However, the maximum dose that a specimen can tolerate is limited by radiation damage. Results from 3D coherent diffraction imaging (CDI) of frozen hydrated specimens have given resolutions of ∼80 nm compared with the expected resolution of 10 nm predicted from theoretical considerations for identifying a protein embedded in water. Possible explanations for this include the inapplicability of the dose-fractionation theorem, the difficulty of phase determination, an overall object-size dependence on the required fluence and dose, a low contrast within the biological cell, insufficient exposure, and a variety of practical difficulties such as scattering from surrounding material. A recent article [Villaneuva-Perez *et al.* (2018), *Optica*, **5**, 450–457] concluded that imaging by Compton scattering gave a large dose advantage compared with CDI because of the object-size dependence for CDI. An object-size dependence would severely limit the applicability of CDI and perhaps related coherence-based methods for structural studies. This article specifically includes the overall object size in the analysis of the fluence and dose requirements for coherent imaging in order to investigate whether there is a dependence on object size. The applicability of the dose-fractionation theorem is also discussed. The analysis is extended to absorption-based imaging and imaging by incoherent scattering (Compton) and fluorescence. This article includes analysis of the dose required for imaging specific low-contrast cellular organelles as well as for protein against water. This article concludes that for both absorption-based and coherent diffraction imaging, the dose-fractionation theorem applies and the required dose is independent of the overall size of the object. For incoherent-imaging methods such as Compton scattering, the required dose depends on the X-ray path length through the specimen. For all three types of imaging, the dependence of fluence and dose on a resolution *d* goes as 1/*d* ^4^ when imaging uniform-density voxels. The independence of CDI on object size means that there is no advantage for Compton scattering over coherent-based imaging methods. The most optimistic estimate of achievable resolution is 3 nm for imaging protein molecules in water/ice using lensless imaging methods in the water window. However, the attainable resolution depends on a variety of assumptions including the model for radiation damage as a function of resolution, the efficiency of any phase-retrieval process, the actual contrast of the feature of interest within the cell and the definition of resolution itself. There is insufficient observational information available regarding the most appropriate model for radiation damage in frozen hydrated biological material. It is advocated that, in order to compare theory with experiment, standard methods of reporting results covering parameters such as the feature examined (*e.g.* which cellular organelle), resolution, contrast, depth of the material (for 2D), estimate of noise and dose should be adopted.

## Abstract 2

The validity of the dose-fractionation theorem and the dependence of required dose on object size and resolution are analysed for various types of X-ray imaging, including coherent-, incoherent- and absorption-based methods. Estimates are given for the achievable resolutions for cells and other soft biological material.

## 1. Introduction   

X-ray images of biological cells often have a low contrast between the diverse contents and the surroundings. This can result in difficulties for imaging because a high fluence (incident photons per unit area) and dose (energy deposited per unit mass in the specimen) is required to obtain the necessary contrast and resolution. However, radiation damage can limit the dose that can be applied. In this article, estimates are given for the contrast and dose achievable for three categories of X-ray imaging. The categories considered are absorption, coherent scattering and incoherent scattering. Coherent scattering can be categorized as methods that require phase determination, perhaps aided by interference effects such as holography, and methods where images are obtained via an objective lens. For the purposes of this article, incoherent scattering includes techniques such as fluorescence measurements (for which the signals add incoherently) and Compton scattering.

For 3D images, some of these methods require a tomographic approach where 2D projections are obtained first. The dose-fractionation theorem (Hegerl & Hoppe, 1976 ▸) states that 3D images can be built up from 2D projection data at a total dose corresponding to that required to see a feature in a 2D projection image.

A 3D reconstruction of a frozen hydrated biological cell was obtained by coherent diffraction imaging (CDI) (Rodriguez *et al.*, 2015 ▸), giving an estimated resolution of 74–99 nm rather than in the 10 nm range predicted by Howells *et al.* (2009 ▸) in a calculation that used the dose-fractionation theorem. In a comments article, Robinson (2015 ▸) investigated possible reasons for this. It was speculated that the dose fractionation might not apply in a simple manner for this case and, following on from that, the scaling of required dose with a resolution *d* might scale as 1/*d* ^5^ rather than the 1/*d* ^4^ seen in the theory. In this case, the resolution *d* is the size of a uniform-density voxel corresponding to a feature to be located.

The dose required to image a feature using CDI was also analysed by Villanueva-Perez *et al.* (2016 ▸) and this was later compared (Villanueva-Perez *et al.*, 2018 ▸) with scanning Compton microscopy (SCM). Both were analysed as 2D projections with the assumption that the dose-fractionation theorem could be applied. The theoretical case for Compton microscopy included an analysis showing that the sample size and resolution dependence of the required dose performed as *W*/*d* ^4^ (given as *L*/*d* ^4^ in the article), where *W* is the thickness of the object and *d* is the resolution. For CDI, the dependence was *W* ^2^/*d* ^6^, where *W* is the width of the sample in two dimensions – a much worse situation despite the fact that, for CDI, the phases were assumed to be known. A *W* ^2^/*d* ^6^ dependence for the required fluence and dose for imaging by CDI would have severe consequences for high-resolution imaging of larger particles by this technique on synchrotrons and perhaps even more on free-electron laser (FEL) sources. For the latter, ptychographic and other scanning techniques will have limited applicability if the aim is to circumvent radiation damage by exploiting the short pulse length of the source. In addition, the flux per pulse is a limitation, particularly for larger specimens where the available photons from an FEL source will be spread out over a larger area when carrying out CDI. Villanueva-Perez *et al.* (2018 ▸) also backed up the theoretical analysis with simulations demonstrating a significant advantage for imaging by SCM compared with CDI for the case of unknown phases. The reasons for the poor apparent performance for CDI (in both theoretical analysis and simulations) are covered in this article.

Both the application of the dose-fractionation theorem and the scaling of required dose with object size and feature size are therefore important issues for the application of imaging methods. The analysis given here is applied to the case of imaging biological cells but some of the principles will apply to imaging other materials at various X-ray energies. A discussion of absorption and phase contrast in X-ray microscopy, together with a comparison with electron microscopy, can be found in the work by Du & Jacobsen (2018 ▸).

## 2. The dose-fractionation theorem   

If an extra feature (a voxel of dimension *d*) replaces a voxel of the same dimension in an object and we calculate the dose required to see this feature in projection, then one should be able to fractionate this dose among an arbitrary number of projections to get a statistically significant 3D image. This is the dose-fractionation theorem (Hegerl & Hoppe, 1976 ▸). Because of the reduced dose, each projection would no longer contain a statistically significant signal showing the feature but the feature would be visible in 3D. The following quotations from relevant articles are given.

‘A statistically significant 3D image can be computed from statistically insignificant projections, as long as the total dose that is distributed among these projections is high enough that it would have resulted in a statistically significant projection, if applied to only one image’, McEwen *et al.* (1995 ▸).

Hoppe & Hegerl (1981 ▸) include ‘The signal-to-noise ratio of this difference is, by the way, independent of the thickness of the specimen’ and ‘The excellent economy of 3D reconstruction is by no means trivial. The important point seems to be that the pieces of information from different sections of the specimen are not only contained in the projections without loss of information, but also that they are coherently added’.

The dose-fractionation theorem was developed to demonstrate that atoms which had a statistically significant contribution in a 2D projection would also be visible in a 3D image at the same accumulated dose. For this case, the contribution in the 2D projection had a defined depth of approximately the size of a single atom. The mathematical basis for the dose-fractionation theorem is not in doubt but there is confusion in the literature about its applicability. For example, it is an incorrect application of the dose-fractionation theorem to claim that a high contrast and resolution in a projection means that the same contrast and resolution with the same overall dose can be obtained in a 3D reconstruction. This would only be the case if the contrast in the 2D image resulted from a feature of depth *d*. Line scans across a projection can give optimistic measurements of the contrast and resolution if, for example, they cross membrane boundaries with the plane of the membrane at an orientation normal to the projection.

The dose-fractionation theorem was originally applied to imaging via electron microscopy. This article analyses the conditions for X-ray imaging under which the required dose-fractionation conditions apply and when the signal given by a feature within the projection is dependent on sample thickness. It is assumed that the aim is to identify a feature of size *d* within an object of size *W*, with both *W* and *d* applying in all three dimensions. Some imaging methods work in projection. In this case, it is assumed for the calculations that the object has a dimension *W* × *W* in the projection and the path length through the object is also *W*. Similarly a depth *d* of the feature contributes to an area of *d* × *d* in the projection.

## 3. Additional considerations for all X-ray imaging techniques   

### 3.1. Attenuation through the sample   

**Figure 1:**
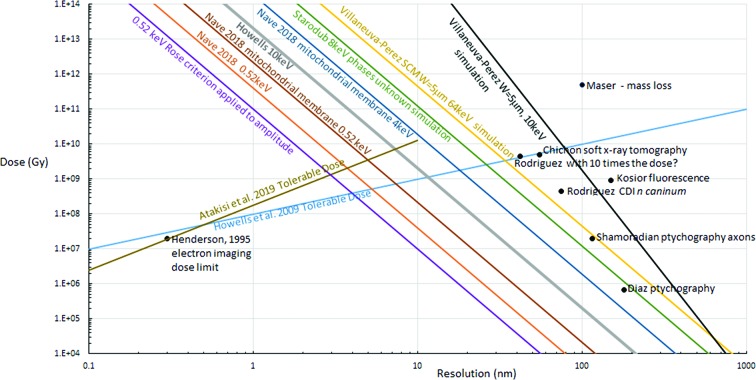
Values for required dose against resolution given in the literature, including this article. For details see Table 1 ▸. The plots are for imaging protein against water/amorphous ice and based on theoretical calculations for CDI unless otherwise indicated. The CDI plots assume a 1/*d* ^4^ resolution dependence of the required dose, except for the Villaneuva-Perez plot which assumes a *W* ^2^/*d* ^6^ dependence. The SCM plot assumes a *W*/*d* ^4^ dependence. Note that the values of *W* for the experiments do not necessarily correspond to the simulations in Villaneuva-Perez *et al.* (2018 ▸). Plots for the resolution dependence for the tolerable dose at which the intensity decreases by half are shown with a 100 MGy nm^−1^ dependence (Howells *et al.*, 2009 ▸) and a *q* ^1.86^ dependence (Atakisi *et al.*, 2019 ▸), where *q* is the wavevector (2π/*d*).

Whether measuring a scattered or absorbed signal, the required fluence and dose have to be corrected by the overall transmission of the object via the term exp(−μ*W*), where *W* is the path length and μ is the absorption coefficient (calculated from atomic composition and density values along the path length). This attenuation length corresponds to a 1/*e* reduction in the intensity and is ∼100 µm for a biological cell at 4000 eV and 2.1–4.5 µm at 520 eV depending on the water content [see Fig. 1 in the work by Nave (2018 ▸)]. As an example, at 510 eV the dose calculated for a thin layer of depth *d* will increase by a factor of 2.7 for a cell of depth 4 µm containing 85% water. In order to make the calculations more general for different-sized objects, the present article does not take account of this correction as it can easily be applied later.

### 3.2. Fluence and dose   

From a fluence *N*_0_, the required dose (*D*) can be calculated using the expression *D* = μ*N*_0_ *E*/ρ where *E* is the energy of an X-ray photon, μ is the absorption coefficient and ρ is the density of the material.

As discussed in the work by Nave (2018 ▸), consideration of the path length of the photoelectrons indicates that this dose should be calculated for values of μ and ρ corresponding to the values for the object as a whole (*e.g.* a biological cell) rather than the feature to be imaged. However, for larger features it is possible that the photoelectrons would remain within the feature. Within the water window, a significant amount of dose could be deposited within the feature for large highly absorbing organelles such as lipid droplets. In addition, the Compton cross section and resultant deposition of energy in the sample have to be included as discussed in the work by Villanueva-Perez *et al.* (2018 ▸). This latter term is excluded from the calculations in this article as, at the X-ray energies considered, dose is mainly dependent on photoelectric absorption.

### 3.3. Contrast and resolution   

The theoretical analysis in this article uses the Rose (1973 ▸) model which defines the contrast in the image. This is also the criterion often used to distinguish low-contrast features in polymers examined by electron microscopy (Libera & Egerton, 2010 ▸). It is assumed that a measurement (*e.g.* electron density, scattered or transmitted photons, fluorescence) *M*_f_ of a feature is to be compared with a measurement *M*_o_ of the surrounding material in the object. The contrast *C* is defined as (*M*_f_ − *M*_o_)/*M*_o_ following Rose (1973 ▸), and the standard deviation in a measurement is σ. The acceptable ratio of the contrast *C* to the noise σ defines the Rose criterion *K* where *K* = *C*/σ. The choice of a particular value for *K* in the final image should depend on the overall sample volume (or area for a 2D projection). Rose (1973 ▸) used an example image which contained 10^5^ pixels. A test spot with a dimension of 1 pixel then had to be recognized as significant against the background and this gives an increased probability of a ‘false alarm’. For 10^5^ pixels (or voxels), a value of *K* = 5.01 would give a similar probability (5000 × 5.5 × 10^−7^) of a false signal as for a single isolated voxel with a value of *K* = 3 (probability = 2.7 × 10^−3^). Rose therefore correctly analysed the dependence of *K* on the number of pixels/voxels. The most appropriate value of the Rose criterion depends on the number of relevant voxels over which the search for a signal occurs. In many cases (*e.g.* filaments and membranes) there is correlation between the density of adjacent pixels meaning that lower values of *K* can be tolerated for individual pixels. An increased value of *K* will require an increase in the dose or fluence, although the increase is only a factor of 2.7 (5.01^2^/3^2^) for an object with 10^5^ pixels/voxels compared with an already identified pixel/voxel. As a value of *K* = 5 has been widely adopted, this is used in this article for consistency with other analyses. Extensions to the Rose model have been covered in several publications (*e.g.* Cunningham & Shaw, 1999 ▸). The Rose model emphasizes the contrast for a voxel of a particular size but is consistent with the resolution defined as the half period of an equal-width line and space grating as discussed, for example, in the work by Schneider (1998 ▸).

Another method for assessing the image is the Fourier shell correlation (FSC) threshold (van Heel & Schatz, 2005 ▸) which addresses the spatial frequency at which there is reliable information content in the data. An argument was made for adopting a 1/2 bit information-content FSC threshold to assess the resolution of the data.

For X-ray imaging, using the Rose criterion in real space, a particular feature with a signal-to-noise ratio (SNR) of 3 has a 0.0027 probability of occurring owing to random noise. The information content of a feature at this level is therefore 8.5 bits (−log_2_0.0027). This is much higher than the typical values used for crystallography and single-particle electron microscopy and reflects the difference in the purpose of the criteria. Using a 1/2 bit FSC threshold for low-contrast X-ray imaging could give an optimistic assessment of image quality. A proper analysis of the relationship between the Rose criterion and FSC thresholds is outside the scope of this article and would need to take account of factors such as the number of image voxels, whether one is in an atomicity or uniform voxel regime and the contrast of features to be identified in the image.

Finally, it is again emphasized that contrast and resolution measurements in 2D projections cannot be simply converted to an expectation for 3D, unless the 2D measurement corresponded to the contrast given by an appropriate depth (*d*) of the feature.

### 3.4. Object size and feature size   

The aim is to identify a feature of size *d* × *d* × *d* within a larger object of size *W* × *W* × *W* with a significance of five times the SNR. In order to illustrate the effects of object size, the calculations in this article are based on the difference between X-ray scattering, absorption or fluorescence for the object with and without the embedded feature. This differs from the approach in the works by Howells *et al.* (2009 ▸) and Nave (2018 ▸) in which the signal for the density difference between a voxel of the feature and an equally sized voxel of the surroundings was analysed.

### 3.5. Feature types and criteria for identification   

A variety of feature types have been used for modelling X-ray imaging via both theoretical considerations and simulations. The signal given by uniform-density spheres or voxels will fall off as ∼1/*d* ^4^ if imaged by coherent scattering (Howells *et al.*, 2009 ▸) or absorption (Nave, 2018 ▸). If they contain ‘atoms’ (Shen *et al.*, 2004 ▸), the scattering will fall off differently (1/*d* ^3^) with resolution. The shape of the resolution element has also been discussed by Starodub *et al.* (2008 ▸) where it is highlighted that for a cubic shape of dimension *d*, the scattering factor would be zero at a scattering vector *q* = 2π/*d*. The signals from Gaussian features have also been evaluated for coherent (Villanueva-Perez *et al.*, 2016 ▸) and incoherent (Villanueva-Perez *et al.*, 2018 ▸) scattering. For coherent scattering, Gaussian features will give a weaker signal at higher resolution compared with uniform-density features and will not contain subsidiary maxima at high resolution. The question then arises regarding which type of feature best represents the components of biological cells at resolutions between 10 and 50 nm. X-ray scattering studies of virus particles (Jack & Harrison, 1975 ▸) show detailed features to a high resolution indicating that, for this type of feature, a Gaussian representation is not appropriate. The theoretical analysis in this article is based on uniform-density voxels although no individual type can represent the wide range of features within biological cells.

## 4. Types of imaging   

The three overall types of imaging are analysed below in terms of the dependence of the required fluence and dose on object size and resolution to obtain sufficient contrast to identify a feature. The following analysis assumes that a feature (*e.g.* a protein molecule) has to be imaged against the surrounding density of an object (*e.g.* the cytosol in a biological cell). Parameters of the feature and object are given the subscripts f and o (*e.g.* *T*_f_ and *T*_o_ for the transmission) while the contrast between them is given the subscript c.

### 4.1. Absorption   

Nave (2018 ▸) expressed the contrast as the difference between the transmission of a single voxel of the object and a single voxel of the feature. In the treatment below, the total thickness of the object is included. The signal for a feature is 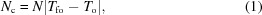
where *N* is the number of incident photons on a voxel, *N*_c_ is the contrast expressed in the number of photons, *T*_fo_ is the transmission for X-rays passing through both the feature (a voxel of dimension *d*) and the object (dimension *W*−*d*), and *T*_o_ is the transmission for X-rays just passing through the object (dimension *W*).

The corresponding standard deviation when comparing the two measurements (*i.e.* for X-rays passing through a feature and those missing the feature) is 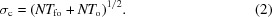
To observe the feature, we require *N*_c_ = *K*σ_c_ where *K* is the Rose criterion. This gives the required incident number of photons as 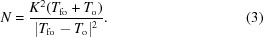
The required fluence is 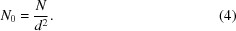
For high transmission values, *T*_fo_ + *T*_o_ ≃ 2 and *T*_fo_ − *T*_o_ is proportional to *d*. This gives *N* proportional to 1/*d* ^2^ [from equation (3)] and *N*_0_ proportional to 1/*d* ^4^ [from equation (4)].

For values of *T*_fo_, *T*_o_ and exp(−μ*W*) near 1, *N*_0_ is independent of the path length *W*. As the path length increases, the exp(−μ*W*) term will eventually lead to a path-length dependence on the required dose as for other forms of X-ray imaging.

At an energy of 520 eV with a 10 nm protein embedded in 200 nm water, the relevant transmissions are those for 200 nm water compared with 190 nm water plus 10 nm protein. The values generated are *T*_pw_ = 0.9664 and *T*_w_ = 0.9784 giving *N* = 3.38 × 10^5^ and *N*_0_ = 3.38 × 10^9^ photons µm^−2^.

### 4.2. Coherent scattering   

Here, coherent scattering refers to the case where the effects of individual features are added coherently. It does not necessarily require a fully coherent beam. The complication for coherently scattered X-rays is whether the phases have to be determined by a reconstruction algorithm or whether they are provided partially or fully by the experimental setup. The ease of phase determination will depend upon the complexity of the image. High-contrast structures (*e.g.* a Siemens Star, gold spheres) should be relatively easy to reconstruct. In this case, the square of the density is similar to the density itself, a situation similar to that where the Sayre (1952 ▸) equation for direct methods in crystallography can be applied. Cellular structures with a higher image entropy will be more comparable with the case for crystallography at lower resolution where direct methods are generally not applicable. However, the oversampling of the data for CDI and related techniques should give a significant advantage at low resolutions compared with the crystallography case. For the theoretical analysis considered here, a large number of photons are assumed to be scattered into each Shannon voxel (see below) with the consequence that the phase-determination step should be robust.

If the phases are precise but the intensities are noisy, this will reflect itself in an image with a poor SNR. This was the case studied from the theoretical point of view by Howells *et al.* (2009 ▸) and Shen *et al.* (2004 ▸). This situation can be simulated by transforming an image, adding noise to the intensities, fixing the phases and then back transforming as investigated by Hagemann & Salditt (2017 ▸). If it does produce a satisfactory image, then, for the cases where the instrument provides phases (*e.g.* via a lens or via some interference technique), the simulation should give a good guidance regarding the possibility of obtaining an image.

The most relevant situation is unknown phases and noisy intensities. Starodub *et al.* (2008 ▸) considered this for CDI and found that a considerable increase in dose and fluence was required compared with the case for known phases. This situation has also been investigated by Villanueva-Perez *et al.* (2016 ▸), Hagemann & Salditt (2017 ▸) and Du *et al.* (2019 ▸). The algorithm now has a much larger search space to investigate and the search space will depend on the size of the object as well as the desired resolution of a feature within it. Although this factor could lead to an object-size dependence for present algorithms, the above simulations were carried out for 2D images. It is probable that 3D data would provide more robust phase determination for CDI.

#### 4.2.1. Coherent diffraction imaging   

The scaling for resolution and sample size has been considered by Villanueva-Perez *et al.* (2016 ▸), Starodub *et al.* (2008 ▸), Robinson (2015 ▸), Shen *et al.* (2004 ▸) and Gureyev *et al.*, 2018 ▸. The issue of detection of a feature within a larger object was also considered by Schropp & Schroer (2010 ▸). Some of these approaches invoke the dose-fractionation theorem when calculating the required fluence/dose, when building up a tomogram from projections. An alternative approach is to consider the statistics in a reciprocal voxel for 3D data with dimensions corresponding to those necessary for Shannon sampling. This will be referred to as a Shannon voxel. Parseval’s theorem states that the root mean square (RMS) density is proportional to the RMS structure-factor amplitude. The uncertainty in the density ρ will therefore follow the uncertainty in the amplitude *A*. In general, both ρ and *A* are complex. Some variants of coherent imaging can derive both the real and imaginary parts of the density. However, to derive the required number of scattered photons, the moduli of the density and amplitude are used. If the requirement is to detect a voxel with a *K*σ_ρ_ change above the surrounding voxels, then 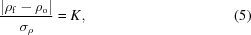
where *K* is the Rose criterion.

We define the contrast as *C* = |ρ_f_ − ρ_o_|/|ρ_o_|. In reciprocal space, where *A* is the amplitude this requires, for each Shannon voxel 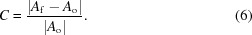
The required statistics for an amplitude measurement in a Shannon voxel at the resolution limit is 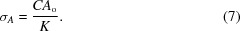
The number of scattered photons *N* is proportional to |*A*_o_|^2^. As σ_*N*_/*N* = 2σ_*A*_/*A* this gives 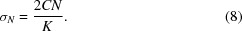
For photon counting, σ_*N*_ = *N* ^1/2^.

The required number of photons in a Shannon voxel is therefore 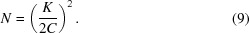
For a Rose criterion of 5 and a contrast of 0.1, this requires 625 photons in each Shannon voxel at the resolution limit. The increased number of Shannon voxels for a larger object is matched by the increased scattering power of the larger object with both being proportional to *W* ^3^. The dose requirement is therefore independent of object size. The dependence of the required dose with resolution (*d*) was recently analysed by Gureyev *et al.* (2018 ▸). For the case of uniform-density voxels considered here, the total scattering from a voxel would be expected to vary as *d* ^3^ corresponding to the number of electrons in the voxel. However, when collecting data for CDI, the required sampling of the data as a function of resolution also needs to be considered. As in crystallography, the correction is given by the Lorentz factor (1/sin 2θ). This can be approximated by 1/*d* for small values of λ/*d*. Combining the two factors, the required fluence and dose will scale as 1/*d* ^4^ in agreement with Howells *et al.* (2009 ▸).

The estimate of 625 photons per Shannon voxel for a contrast of 0.1 is consistent with the value of 6.25 photons for an isolated feature (Starodub *et al.*, 2008 ▸) where the Rose criterion is applied to the modulus of the scattered amplitude rather than the intensity. The above discussion is based on CDI but related techniques such as phase-contrast projection imaging, Zernike phase contrast, holographic imaging and ptychography should follow a similar behaviour if phases are known.

Away from an absorption edge, the real part δ of the refractive index varies approximately with wavelength as λ^2^ whereas the imaginary part β varies as λ^4^. The scattering depends mainly on δ whereas the absorption (responsible for radiation damage) depends on β. It is sometimes stated that this means there is a significant signal-to-dose advantage when using shorter wavelength X-rays when carrying out coherence-based imaging techniques. However, there are a number of other factors that need to be taken in to consideration including the fact that high-energy photons deposit more energy when absorbed. The dose-to-signal ratio has a very weak wavelength dependence at energies above the water window as illustrated by the corresponding graphs in the works by Schneider (1998 ▸) and Howells *et al.* (2009 ▸). It is also consistent with the energy dependence of signal to dose for crystallography as shown in Fig. 2 of Cowan & Nave (2008 ▸), a reassuring situation that allows for analysis of mixtures of crystalline and non-crystalline material at different wavelengths.

#### 4.2.2. Other coherent-scattering techniques   

Two other coherent-scattering techniques are considered in order to provide some validation for the conclusions about coherent X-ray scattering in the previous section.[Sec sec4.2.1]

Henderson (1995 ▸, sections 3 and 4) includes comparisons between various sorts of phase-contrast microscopy. It also includes calculations for single-particle imaging by electron microscopy showing that the number of images required to get a particular resolution is independent of particle size (Table 1, column O). The argument used is similar to the one used here for CDI. The signal per object (in this case, a protein molecule) is greater in projection for larger particles but more projections are required for the larger objects.

In the case of electron microscopy of single particles at near atomic resolution, the assumption of a voxel of uniform density does not apply. The scaling of required dose with resolution performed as 1/*d* ^3^ as described for X-ray imaging in the works by Shen *et al.* (2004 ▸) and Gureyev *et al.* (2018 ▸).

For crystallography there is an amplification factor given by the number of unit cells in the crystal. Assuming the same space group (*e.g.* *P*1) and with the same number of unit cells, this amplification factor will be the same for a small molecule or a larger one. The crystal size will be proportionally larger for the larger molecule. We want to obtain the same resolution from both (*e.g.* to see atoms or atomic groups such as amino acids). The bigger crystal (and molecule) will require the same fluence (and dose) as the smaller one. The number of photons required to do this will be greater for the larger crystal containing larger protein molecules but they will be spread out over a bigger volume giving the same required dose. Crystallography normally aims to identify atoms and groups of atoms rather than image uniform voxels. In this case, the required dose and fluence has a 1/*d* ^3^ dependence as described by Shen *et al.* (2004 ▸). For very high resolution studies where the aim is to image charge density, the dose dependence should trend towards 1/*d* ^4^ behaviour.

### 4.3. Incoherent scattering and fluorescence   

Incoherent X-ray scattering normally refers to Compton scattering. The analysis here is given for X-ray fluorescence. Neglecting techniques such as fluorescence holography, this is also an incoherent process. Measuring X-ray fluorescence via scanning fluorescence X-ray microscopy is a well established technique for identifying concentrations of metal (and other fluorescing) atoms in biological cells. In contrast to the case described in Section 4.1 where differences in the transmitted beam are measured, the signal for fluorescence imaging is proportional to the absorbed photons. In this analysis, it is assumed that an object with dimensions *W* (in each direction) contains a concentration ρ_o_ of a particular metal atom. A particular feature of size *d* contains a higher concentration ρ_f_. The sample is scanned with a beam of dimension *d* in each direction, thus defining the minimum resolution obtainable from a feature of dimension *d*.

To satisfy the Rose criterion we need 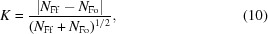
where *N*_Fo_ and *N*_Ff_ are the number of fluorescent photons produced from the beam incident on the bulk of the object alone and those also incident on the feature.

The number of fluorescent photons from the bulk is 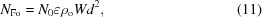
where *N*_0_ is the fluence and ∊ is an excitation factor corresponding to the probability of fluorescence from the relevant atoms with a total mass *W* *d* ^2^ρ_o_ within the beam.

The number of fluorescent photons from a beam which encounters a feature within the bulk is 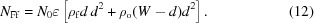
This gives 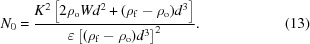
For the case where there is little fluorescent contribution from the bulk, *W*ρ_o_ is small and the required fluence performs as 1/*d* ^3^. For the case where *W*ρ_o_ >> *d*ρ_f_, 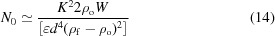
and the required fluence performs as *W*/*d* ^4^.

The SNR in this case therefore depends on sample thickness for the same feature size but, when this is taken into account, the dose-fractionation theorem still applies. It requires a greater dose to identify a feature within a thicker specimen but, if this feature is visible in a single projection, the dose can be fractionated among the required number of projections for tomography.

The value of ∊ depends on factors such as the probability of absorption, transition probability, fluorescence yield and jump factor. The latter two are discussed in the work by Brunetti *et al.* (2004 ▸) with accompanying tables.

A general expression for determining the excitation factor is 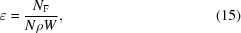
where *N* (= *N*_0_ *d* ^2^) is the total number of photons incident on the specimen, *N*_F_ is the number of fluorescent photons and ρ is the concentration of the atom of interest. An example of calculating ∊ for potassium is given below. Using the tools on the CXRO website (Henke *et al.*, 1993 ▸) for the potassium edge, with ρ = 0.1 g cm^−3^ (10^−13^ g µm^−3^) and *W* = 1 µm, a transmission of 0.99861 at 3600 eV and 0.98696 at 3610 eV is calculated with the difference corresponding to the jump factor for the *K* absorption edge. The number of absorbed photons at the *K* edge is therefore 0.0116 of the incident photons (*N*) with 0.14 of these producing fluorescent photons at the *K*_α_ and *K*_β_ lines (Brunetti *et al.*, 2004 ▸), giving a value of *N*_F_/*N* = 0.00163 and ∊ = 1.631 × 10^13^ g µm^−2^.

The above analysis is similar to that given for Compton imaging in the work by Villanueva-Perez *et al.* (2018 ▸) (including the appendix). These authors concluded that the scaling for the required dose follows *W*/*d* ^4^ (given as *L*/*d* ^4^ in the article).

## 5. Comparison of dose and resolution estimates   

Many of the methods involve imaging by more than one technique. Coherent-based methods such as CDI, holography and ptychography can be used to obtain the complex refractive index. Zernike methods also depend on both phase and absorption contrast while SCM can obtain a signal from both coherent and incoherent scattering. The most appropriate technique will depend on factors such as the thickness of the specimen as well as the contrast mechanism.

Table 1 ▸ gives a comparison of fluence and dose estimates obtained from this article and the literature for identifying features within biological cells. Different assumptions are present for the various analyses. For example, in the works by Howells *et al.* (2009 ▸) and Nave (2018 ▸) the assumption was that, for coherent imaging, a value of *K* = 5 applied to the intensity is required in each relevant reciprocal volume. Starodub *et al.* (2008 ▸) suggested that a value of 2 × 5^1/2^ (corresponding to a distribution of the number of photons with an RMS value of 6.25 rather than 25) was sufficient as the image is derived from the modulus of the scattered amplitude rather than the intensity. If this is applied, the dose and fluence figures given by Nave (2018 ▸) and Howells *et al.* (2009 ▸) for phase contrast would be a factor of four lower, or improvements in resolution would be obtained for the same dose. These values are from analytical calculations with known phases. Values of dose and fluence from Starodub *et al.* (2008 ▸) using numerical simulations with unknown phases in 2D are given for comparison. In addition, Starodub *et al.* (2008 ▸) also discussed the dependence of required fluence on the type of feature to be examined. Most of the examples in Table 1 ▸ use uniform-density voxels but 2D Gaussian features were used for the numerical simulations by Villanueva-Perez *et al.* (2018 ▸). Where the same approach is used, the table provides guidance for the dependence on parameters such as sample contrast and energy dependence. With these parameters fixed, the table provides guidance on the different approaches (*e.g.* with known phases or unknown phases).

It is seen that much lower doses are required for imaging low-density high-carbon-content components in the water window compared with imaging at higher energy. The combination of absorption and phase contrast has been analysed in terms of ‘optimum phase contrast’ (Schneider, 1998 ▸) in the context of imaging protein against water with a Zernike phase plate. Imaging within the water window also requires much lower doses for the cellular components heterochromatin, mitochondrial inner membrane, lipid and, to a lesser extent, high-density starch granules. X-ray imaging at higher energies has been shown to be reasonably successful in 2D imaging of high-density cellular components such as chloro­plasts, pyrenoids and polyphosphate bodies (Deng *et al.*, 2017 ▸).

At energies above the water window there are two reasons why the required dose is significantly higher than at energies within the water window. Firstly, the contrast difference between carbon-containing components and water decreases above the water window. Some biological membranes, if lightly loaded with protein, could actually match the cytosol density, effectively making them invisible at higher energies, whereas their high carbon content would make them very visible at 520 eV. Secondly, the water (typically corresponding to 70 to 85% of the cell) absorbs much more strongly at energies above the oxygen absorption edge, therefore increasing the dose for the same fluence.

Fig. 1 ▸ indicates the positions of some of the examples given in Table 1 ▸ on a dose versus resolution plot. In addition, the Howells *et al.* (2009 ▸) model of the dose threshold as a function of resolution is shown together with a more recent one (Atakisi *et al.*, 2019 ▸) obtained from radiation-damage studies of protein crystals. Similar diagrams occur in the works by Shen *et al.* (2004 ▸), Howells *et al.* (2009 ▸) and Villanueva-Perez *et al.* (2018 ▸). The Atakisi radiation-damage model assumes that local disordering reactions occur. If these applied during a tomographic data collection for X-ray imaging the result would be a loss of resolution as well as possible artefacts. The highest attainable resolution shown in Fig. 1 ▸ is that for coherent imaging at 0.52 keV for protein contrasted against water, assuming the Rose criterion (*K* = 5) applied to the amplitude in a Shannon voxel and the Atakisi *et al.* (2019 ▸) model of tolerable dose applied. All these conditions, plus the assumption of efficient phase determination, would have to be met to realize the 3 nm resolution implied in Fig. 1 ▸. A 3 nm focal spot is beyond the capabilities of present X-ray optics and this implies that ptychography in the far field is likely to be necessary in order to approach such resolutions. The dose-limited resolution would in any case degrade to ∼5 nm for a 10 µm thick sample owing to the attenuation of the signal at 0.52 keV.

The example used by Villanueva-Perez *et al.* (2018 ▸) to compare SCM and CDI consisted of a 34 nm feature embedded in a 5 µm sized object and assumed that the dose dependence performed as *W*/*d* ^4^ for SCM (given as *L*/*d* ^4^ in the article) and as *W* ^2^/*d* ^6^ for CDI. The theoretical analysis then showed that the required dose for CDI would be ∼10^3^ higher than for SCM. If a 1/*d* ^4^ dependence for CDI applied, the dose required for CDI would instead be ∼20 times less than for SCM. The reason for the strong particle size and resolution dependence for CDI in the work by Villanueva-Perez *et al.* (2016 ▸) comes from an assumption in the theoretical section. Equation (9) in this section gives the required SNR (using the nomenclature in the present article) as 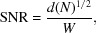
where *N* is the total number of scattered photons from a pixel of size *d* × *d*. The analysis was carried out in two dimensions with the assumption that the dose-fractionation theorem applied. The *d*/*W* term corresponds to the Shannon sampling requirement in each dimension. The expression for the SNR is equivalent to requiring that each pixel (size *d* × *d*) in the object contributes the same number of scattered photons to each Shannon pixel in the scattering pattern and this is independent of the object size. No justification is provided for this and the *W* ^2^/*d* ^6^ dose dependence is a consequence of this assumption. In coherent imaging, the complex amplitudes from the scattering of the pixels or voxels are added before squaring for the intensity. This preserves the scattering statistics from each pixel or voxel in the presence of the surrounding ones and leads to the 1/*d* ^4^ dependence.

The simulations (Villanueva-Perez *et al.*, 2018 ▸) appeared to show an advantage for SCM compared with CDI. These simulations were carried out for Gaussian features in 2D. Other 2D simulations show that up to two orders of magnitude increase in dose can be required for CDI compared with the situation where the phases are known (*e.g.* Starodub *et al.*, 2008 ▸). The advantages of Compton microscopy over CDI therefore depend on whether a larger dependence (*e.g.* *W* ^2^/*d* ^6^ as in the work by Villanueva-Perez *et al.*, 2018 ▸) for coherent-scattering techniques is valid for the case of unknown phases. There is no theoretical basis for this and the performance of CDI in the simulations might have been because of the less-robust phasing procedures with 2D data and/or the use of Gaussian features. In general, coherent-scattering methods should be more powerful than incoherent-scattering methods. For coherent scattering, the phase relationships between waves scattered by each component are retained as opposed to a simple summing of the intensities.

Near-field holography appears to offer advantages for phase retrieval over CDI (Hagemann & Salditt, 2017 ▸; Du *et al.*, 2019 ▸). Ptychography can also provide some phase information because of the overlap between probe positions (Bunk *et al.*, 2008 ▸). This was confirmed in the work by Du *et al.* (2019 ▸), where an advantage was found for ptychography over holography. These simulations were carried out in 2D and assumed that only phase contrast was present and that the illumination function was known. For the general case, with no assumptions about the specimen, there are four unknowns corresponding to the phase and amplitude for both the sample scattering and the incident beam. Near-field ptychography (Stockmar, 2013 ▸; Clare *et al.*, 2015 ▸) aims to address these four unknowns. Far-field ptychography could also determine these four unknowns by exploiting the overlap between the probe positions. Use of structured or diffuse illumination might offer a further advantage (Liu *et al.*, 2008 ▸). Further development of reconstruction algorithms together with simulations and experiments on realistic samples should hopefully resolve the most appropriate technique for coherent-based imaging. For synchrotron-radiation sources, techniques such as ptychography and near-field holography appear to be replacing CDI. However, if the aim is to outrun radiation damage by exploiting the short pulse length, ptychography has limitations on FELs. This is because radiation-damage effects can cover an area larger than the initial illumination.

For coherent-based methods, the Rose criterion should be applied to the modulus of the amplitude (as in the work by Starodub *et al.*, 2008 ▸) of the scattered wave rather than to the intensity. However, at any radiation-damage limit, this would only lead to a small improvement in resolution (*e.g.* from 10 to 7.5 nm) because of the strong dependence of required dose with resolution and the dependence of the radiation-damage limit on resolution.

Methods which utilize an objective lens or involve scanning a small focal spot at the specimen position can suffer from depth-of-focus limitations. As an example, Villanueva-Perez *et al.* (2018 ▸) estimate the depth of focus of their proposed SCM as 10 µm at 64 keV with a focal spot of 10 nm. Methods are being developed to handle depth-of-focus (or depth-of-field) effects (*e.g.* Tsai *et al.*, 2016 ▸; Gilles *et al.*, 2018 ▸) but depth-of-focus effects are likely to lead to a decrease in the information content of the data for incoherent-based methods. The other disadvantage of incoherent-scattering methods is the path-length dependence of the signal, meaning that thicker specimens will require a higher dose to achieve the same SNR as thinner specimens. The two effects together will limit the application of fluorescence microscopy as the required dose will increase for thicker specimens if the *W*ρ_o_ term is significant.

It is of interest to compare the dose required for imaging a typical cellular feature by fluorescence with that required for imaging via ptychography at the same resolution. With the potassium concentrations used in the work by Nave (2018 ▸), the fluence and dose required to image the potassium in heterochromatin (potassium concentration of 0.018 g cm^−3^) at 30 nm resolution against 5 µm of the surrounding nucleosol (0.007 g cm^−3^) can be calculated. The required fluence is 1.09 × 10^12^ photons µm^−2^ and the required dose is 7.60 × 10^9^ Gy. These values assume that all the fluorescent photons can be collected. The estimate would increase to 6 × 10^11^ Gy at a resolution of 10 nm. This is much greater than the required dose to image the heterochromatin itself at either 4 keV or 0.52 keV by coherent-imaging methods (see Table 1 ▸). This is consistent with the significant damage seen in phase-contrast images after a fluorescence scan (Kosior *et al.*, 2012 ▸, 2013 ▸). For situations where there is a high concentration of metal atoms in the feature, where the specimen is thinner and where the surrounding material contains a much lower concentration, the required dose will of course be much lower. A discussion of radiation damage in scanning-probe fluorescence microscopy can be found in the work by Jonge *et al.* (2014 ▸).

## 6. Conclusions   

Assuming known phases for coherent scattering, the required dose and fluence for both coherent scattering and absorption follows a 1/*d* ^4^ dependence, where *d* is the resolution, and a 1/*C* ^2^ dependence, where *C* is the contrast. For incoherent scattering, the required dose and fluence follows a *W*/*d* ^4^ dependence if there is a large signal from the bulk of the object and a 1/*d* ^3^ dependence if the signal from the bulk is negligible. The tolerable dose is much less well determined with possibilities ranging from a 1/*d* dependence (Howells *et al.*, 2009 ▸) to a higher power dependence, *e.g.* 1/*d* ^1.86^ as suggested by Atakisi *et al.* (2019 ▸).

The present article is not in agreement with the theoretical dose dependence (*W* ^2^/*d* ^6^) for CDI with known phases given in the works by Villanueva-Perez *et al.* (2018 ▸, 2016 ▸). The simulations in these articles are carried out in 2D, use Gaussian features and do not cover a range of values of *W* and *d*, so only provide a partial test of the theoretical model. It is possible that the lower resolution obtained in the work by Rodriguez *et al.* (2015 ▸) was caused by a combination of factors. Firstly, the cellular components could have had a much lower contrast than that given by a protein molecule in pure water (or amorphous ice). Secondly, the phase-determination step is unfavourable for CDI, compared for example with ptychography and near-field holography. Thirdly, the specimen exposure (fluence) was far below that required for higher-resolution imaging and also below that for any radiation-damage threshold at the resolution obtained. If the dose was increased by a factor of 10, a resolution of 42 nm might have been obtained. The dose would then have been near the radiation-damage limit according to the Howells *et al.* (2009 ▸) model but less than that for the Atakisi *et al.* (2019 ▸) model (see Fig. 1 ▸). The radiation-damage models for X-ray imaging do not appear to have been tested in detail over a wide range of resolutions. Mass loss was observed at 100 nm resolution with a dose of 5 × 10^11^ Gy (Maser *et al.*, 2000 ▸) but this does not correspond to the half-dose metric shown in Fig. 1 ▸. In addition, the relationship between radiation damage for crystalline specimens and that for non-crystalline specimens would also merit further investigation. It is possible that the damage would be similar if only local disorder occurred rather than longer-range disorder (Atakisi *et al.*, 2019 ▸).

Measurements in the water window offer a considerable advantage for imaging low-density components in frozen hydrated biological cells because of the increased phase and absorption contrast. If phase retrieval is reasonably efficient there will be an advantage in lensless imaging methods over those based on objectives lenses because of inefficiency and depth-of-focus issues for objective lenses. For thicker specimens, again, provided phase retrieval is reasonably efficient, coherent-imaging methods will offer a significant advantage compared with incoherent methods such as SCM. Given these assumptions, there is no dependence of the required dose with object size until significant X-ray attenuation occurs. Further simulations and tests on realistic samples are obviously required to validate these conclusions. Significant developments are occurring in electron microscopy for thicker samples including techniques such as tomographic imaging, serial sectioning and focused ion-beam milling, for both imaging cells and brain tissue. For a discussion of these electron-microscopy techniques see, for example, Xu *et al.* (2017 ▸) and Hoffman *et al.* (2020 ▸). A detailed comparison of the relative abilities of imaging by electrons and X-rays can be found in the work by Du & Jacobsen (2018 ▸).

The above discussion applies to frozen hydrated specimens and assumes that this is the nearest practical approach to a native state when using ionizing radiation to obtain images. This is undoubtedly the case for imaging at near-atomic resolution for protein structure determination but there are a large number of electron-imaging studies using other forms of specimen treatment such as freeze substitution, fixation, resin embedding and staining. If these specimen-preparation techniques are considered to be acceptable at resolutions achievable by X-ray imaging and they offer enhanced contrast, this would improve the capabilities of X-ray imaging. This issue needs to be considered along with the usefulness of imaging biological cells at the relatively low resolution obtainable by X-ray imaging. Many of the processes within biological cells take place at a local level where the interest is in examining the interactions involving individual macromolecules and there is a significant advantage with the higher resolution obtained with electron imaging. X-ray imaging at higher energies is likely to have a significant role for thicker samples such as biological tissue and organoids. For imaging the neurons and synapses in the brain, the interactions of interest could take place over large distances so meaningful data for large objects would be required and potentially available via X-ray imaging. A demonstration of the capabilities is the imaging of axons in mouse-brain tissue (Shahmoradian *et al.*, 2017 ▸) examined at cryotemperatures, prepared using chemical fixation and a 1.18 *M* sucrose cryoprotectant (density is ∼1.23 g cm^−3^), and examined by ptychograpy. The mass densities ranged from ∼0.99 to a maximum of 1.42 g cm^−3^ consistent with there being adequate contrast to image the features. The contrast was attributed to the high density of the cryoprotectant compared with the density of the myelin. Some details of the data collection are given in Table 1 ▸, and the dose and resolution values are shown in Fig. 1 ▸.

Whether X-ray imaging of intrinsically low-contrast bio­logical specimens can reach the most optimistic estimates of achievable resolution (*e.g.* 3 nm) depends on a variety of factors. These include which radiation-damage model is correct, the reliability of any phase-retrieval algorithm, the (energy-dependent) contrast for the features of interest, the statistical precision required, the acceptability of contrast-enhancing specimen-preparation procedures and the overall efficiency of the instrument. A systematic study of many of these factors is necessary in order to give a more reliable estimate of the potential of X-ray imaging for these type of materials. At present, there are few observations which can be used to populate diagrams such as Fig. 1 ▸ in this article. In order to compare theory or simulations with experiment, standard methods of reporting results covering parameters such as the feature examined (*e.g.* which cellular organelle), resolution, contrast, depth of the material (for 2D), estimate of noise and dose should be encouraged. Much effort has gone into implementing this for fields such as macromolecular crystallography but it has to be admitted that this is still an ongoing process.

## Figures and Tables

**Table 1:** A comparison of the required fluences and doses for X-ray imaging NA = not applicable. Feature acronyms: heterochromatin (HC), inner mitochondrial membrane (IMM), lipid droplet neutral core (LDNC), starch granule (SG) and heterochromatin potassium [HC(K)]. Data that appear in bold are used for the plots in Fig. 1 ▸. Where a resolution other than 10 nm is given for the theory and simulations, additional estimates are provided assuming 10 nm resolutions, using the theoretical model.

Reference	Contrast	Feature	Object	Energy (keV)	*d* (nm)	*W* (µm)	Fluence (photons µm^−2^)	Dose (Gy)
Theoretical studies (assume known phases for coherence-based methods)								
Nave (2019 ▸)†	Absorption	Protein	Water	0.52	10	NA	3.29 × 10^9^	1.01 × 10^8^
Nave (2019 ▸)†	Absorption	Protein	Water	4	10	NA	1.14 × 10^15^	6.78 × 10^12^
Schneider (1998) ▸ ‡	Absorption	Protein	Water	0.52	30	10	4.00 × 10^7^	2.00 × 10^6^
Schneider (1998) ▸ ‡	Absorption	Protein	Water	0.52	10	10	3.24 × 10^9^	1.62 × 10^8^
**Nave (2018 ▸)†**	**Phase**	**Protein**	**Water**	**0.52**	**10**	**NA**	**1.31 × 10^9^**	**4.01 × 10^7^**
Nave (2018 ▸)†	Phase	Protein	Water	4	10	NA	3.30 × 10^11^	1.96 × 10^9^
**Howells *et al.* (2009) ▸**	**Phase**	**Protein**	**Water**	**10**	**10**	**NA**	**2.00 × 10^12^**	**2.00 × 10^9^**
Schneider (1998) ▸ ‡	Phase (Zernike)	Protein	Water	0.52	30	10	1.00 × 10^7^	5.00 × 10^5^
Schneider (1998) ▸ ‡	Phase (Zernike)	Protein	Water	0.52	10	10	8.10 × 10^8^	4.05 × 10^7^
**This article§**	**Phase**	**Protein**	**Water**	**0.52**	**10**	**NA**	**3.27 × 10^8^**	**1.00 × 10^7^**
This article	Fluorescence	HC (K)	Nucleosol	3.61	30	5	1.09 × 10^12^	7.60 × 10^9^
This article	Fluorescence	HC (K)	Nucleosol	3.61	10	5	8.82 × 10^13^	6.15 × 10^11^
Nave (2019 ▸)†	Absorption	HC	Nucleosol	0.52	10	NA	5.77 × 10^10^	2.09 × 10^9^
Nave (2019 ▸)†	Absorption	IMM	Cytosol	0.52	10	NA	1.71 × 10^10^	5.23 × 10^8^
Nave (2019 ▸)†	Absorption	LDNC	Cytosol	0.52	10	NA	1.03 × 10^10^	3.15 × 10^8^
Nave (2019 ▸)†	Absorption	SG	Cytosol	0.52	10	NA	3.83 × 10^10^	1.17 × 10^9^
Nave (2018 ▸)†	Phase	HC	Nucleosol	0.52	10	NA	2.24 × 10^10^	6.85 × 10^8^
**Nave (2018 ▸)†**	**Phase**	**IMM**	**Cytosol**	**0.52**	**10**	**NA**	**7.13 × 10^9^**	**2.18 × 10^8^**
Nave (2018 ▸)†	Phase	LDNC	Cytosol	0.52	10	NA	5.50 × 10^9^	1.68 × 10^8^
Nave (2018 ▸)†	Phase	SG	Cytosol	0.52	10	NA	6.30 × 10^9^	1.93 × 10^8^
Nave (2018 ▸)†	Phase	HC	Nucleosol	4	10	NA	3.81 × 10^12^	2.27 × 10^10^
**Nave (2018 ▸)†**	**Phase**	**IMM**	**Cytosol**	**4**	**10**	**NA**	**3.18 × 10^12^**	**1.89 × 10^10^**
Nave (2018 ▸)†	Phase	LDNC	Cytosol	4	10	NA	2.40 × 10^12^	1.43 × 10^10^
Nave (2018 ▸)†	Phase	SG	Cytosol	4	10	NA	4.32 × 10^11^	2.56 × 10^9^

Simulations (unknown starting phases for coherence-based methods)								
**Starodub *et al.* (2008 ▸)¶**	**Phase**	**Protein**	**Water**	**10**	**10**	**NA**	**1.20 × 10^14^**	**1.20 × 10^11^**
**Villanueva-Perez *et al.* (2018) ▸††**	**Phase**	**Protein**	**Water**	**10**	**34**	**5**	**1.43 × 10^15^**	**1.16 × 10^12^**
**Villanueva-Perez *et al.* (2018) ▸††**	**Phase**	**Protein**	**Water**	**10**	**10**	**5**	**2.21 × 10^18^**	**1.79 × 10^15^**
**Villanueva-Perez *et al.* (2018) ▸††**	**Compton**	**Protein**	**Water**	**64**	**34**	**5**	**1.15 × 10^14^**	**3.63 × 10^9^**
**Villanueva-Perez *et al.* (2018) ▸††**	**Compton**	**Protein**	**Water**	**64**	**10**	**5**	**1.53 × 10^16^**	**4.84 × 10^11^**

Experimental results from biological cells								
**Rodriguez *et al.* (2015) ▸**	**CDI**	**Various**	**Cytosol**	**8**	**75**	**∼1–5**	**—**	**4.55 × 10^8^**
**Shahmoradian *et al.* (2017) ▸**	**Ptychography**	**Axons**	**1.8 *M* sucrose**	**6.2**	**115**	**∼40**	**—**	**2.0 × 10^7^**
**Diaz *et al.* (2015) ▸**	**Ptychography**	**Various**	**Cytosol**	**6.2**	**180**	**—**	**—**	**6.7 × 10^5^**
**Kosior *et al.* (2012 ▸, 2013 ▸)‡‡**	**Fluorescence K, Zn, *etc*.**	**Various**	**Cytosol**	**17**	**150**	**—**	**—**	**9 × 10^8^**
**Chichón *et al.* (2012) ▸§§**	**Absorption, full field**	**Various**	**Cytosol**	**0.51**	**55**	**—**	**—**	**5.0 × 10^9^**
**Maser *et al.* (2000) ▸¶¶**	**Absorption, STXM**	**Various**	**Cytosol**	**0.52**	**100**	**—**	**—**	**5.0 × 10^11^**

† The calculation of dose assumes the energy of the absorbed photons is spread throughout the object rather than just within the feature.

‡ Estimated from Figs. 5 and 7. Assumes attenuation through an ice-layer thickness of 10 µm.

§ As for row 5 but with the Rose criterion applied to the amplitude rather than the intensity giving a factor of four reduction in fluence and dose.

¶ Dark line, Fig. 7.

†† Tables 1 and 2. Note that there appears to be a mistake for the density of the biomolecule in Table 1 which should presumably be 1.35 g cm^−3^. The 34 nm value for *W* corresponds to 2σ for a Gaussian feature.

‡‡ Scanning fluorescence X-ray microscopy in 2D. The probe size is 150 nm.

§§ Soft X-ray tomography with zone-plate objective (the efficiency is ∼10%).

¶¶ Scanning transmission X-ray microscopy (STXM) observation. The dose figure is that at which observable mass loss occurs.

## References

[bb1] Atakisi, H., Conger, L., Moreau, D. W. & Thorne, R. E. (2019). *IUCrJ*, **6**, 1040–1053. 10.1107/S2052252519008777 PMC6830208 31709060

[bb2] Brunetti, A., Sanchez del Rio, M., Golosio, B., Simionovici, A. & Somogyi, A. (2004). *At. Spectrosc.* **59**, 1725–1731.

[bb3] Bunk, O., Dierolf, M., Kynde, S., Johnson, I., Marti, O. & Pfeiffer, F. (2008). *Ultramicroscopy*, **108**, 481–487. 10.1016/j.ultramic.2007.08.003 17764845

[bb4] Chichón, F. J., Rodríguez, M. J., Pereiro, E., Chiappi, M., Perdiguero, B., Guttmann, P., Werner, S., Rehbein, S., Schneider, G., Esteban, M. & Carrascosa, J. L. (2012). *J. Struct. Biol.* **177**, 202–211. 10.1016/j.jsb.2011.12.001 PMC7119024 22178221

[bb5] Clare, R. M., Stockmar, M., Dierolf, M., Zanette, I. & Pfeiffer, F. (2015). *Opt. Express*, **23**, 19728. 10.1364/OE.23.019728 26367630

[bb6] Cowan, J. A. & Nave, C. (2008). *J. Synchrotron Rad.* **15**, 458–462. 10.1107/S0909049508014623 18728316

[bb7] Cunningham, I. & Shaw, R. (1999). *J. Opt. Soc. Am. A*, **16**, 621.

[bb8] Deng, J., Vine, D., Chen, S., Jin, Q., Nashed, Y., Peterka, T., Vogt, S. & Jacobsen, C. (2017). *Sci. Rep.* **7**, 445. 10.1038/s41598-017-00569-y PMC5428657 28348401

[bb9] Diaz, A., Malkova, B., Holler, M., Guizar-Sicairos, M., Lima, E., Panneels, V., Pigino, G., Bittermann, A., Wettstein, L., Tomizaki, T., Bunk, O., Schertler, G., Ishikawa, T., Wepf, R. & Menzel, A. (2015). *J. Struct. Biol.* **192**, 461–469. 10.1016/j.jsb.2015.10.008 26470812

[bb10] Du, M., Gursoy, D. & Jacobsen, C. (2019). arXiv:1908.06770.

[bb11] Du, M. & Jacobsen, C. (2018). *Ultramicroscopy*, **184**, 293–309. 10.1016/j.ultramic.2017.10.003 PMC5696083 29073575

[bb12] Gilles, M., Nashed, Y., Du, M., Jacobsen, C. & Wild, S. (2018). *Optica.* **5**, 1078–1086. 10.1364/OPTICA.5.001078 PMC6217975 30406160

[bb13] Gureyev, T. E., Kozlov, A., Nesterets, Y. I., Paganin, D. M., Martin, A. V. & Quiney, H. M. (2018). *IUCrJ*, **5**, 716–726. 10.1107/S2052252518010941 PMC6211529 30443356

[bb14] Hagemann, J. & Salditt, T. (2017). *J. Appl. Cryst.* **50**, 531–538. 10.1107/S1600576717003065 PMC5377347 28381977

[bb15] Heel, M. van & Schatz, M. (2005). *J. Struct. Biol.* **151**, 250–262. 10.1016/j.jsb.2005.05.009 16125414

[bb16] Hegerl, R. & Hoppe, W. (1976). *Z. Für Naturforsch. A*, **31**, 1717–1721.

[bb17] Henderson, R. (1995). *Quart. Rev. Biophys.* **28**, 171–193. 10.1017/s003358350000305x 7568675

[bb18] Henke, B., Gullikson, E. & Davis, J. (1993). *At. Data Nucl. Data Tables*, **54**, 181–342.

[bb19] Hoffman, D., Shtengel, G., Xu, C., Campbell, K., Freeman, M., Wang, L., Milkie, D., Pasolli, H., Iyer, N., Bogovic, J., Stabley, D. R., Shirinifard, A., Pang, S., Peale, D., Schaefer, K., Pomp, W., Chang, C., Lippincott-Schwartz, J., Kirchhausen, T., Solecki, D. J., Betzig, E. & Hess, H. F. (2020). *Science*, **367**, eaaz5357. 10.1126/science.aaz5357 PMC7339343 31949053

[bb20] Hoppe, W. & Hegerl, R. (1981). *Ultramicroscopy*, **6**, 205–206.

[bb21] Howells, M., Beetz, T., Chapman, H., Cui, C., Holton, J., Jacobsen, C., Kirz, J., Lima, E., Marchesini, S., Miao, H., Sayre, D., Shapiro, D. A., Spence, J. C. H. & Starodub, D. (2009). *J. Electron Spectrosc. Relat. Phenom.* **170**, 4–12. 10.1016/j.elspec.2008.10.008 PMC2867487 20463854

[bb22] Jack, A. & Harrison, S. C. (1975). *J. Mol. Biol.* **99**, 15–25. 10.1016/s0022-2836(75)80155-0 173853

[bb23] Jonge, M. D. de, Ryan, C. G. & Jacobsen, C. J. (2014). *J. Synchrotron Rad.* **21**, 1031–1047. 10.1107/S160057751401621X PMC4151681 25177992

[bb24] Kosior, E., Cloetens, P., Devès, G., Ortega, R. & Bohic, S. (2012). *Appl. Phys. Lett.* **101**, 263102.

[bb25] Kosior, E., Cloetens, P., Devès, G., Ortega, R. & Bohic, S. (2013). *Appl. Phys. Lett.* **102**, 109901.

[bb26] Libera, M. & Egerton, R. (2010). *Polym. Rev.* **50**, 321–339.

[bb27] Liu, Y., Chen, B., Li, E., Wang, J., Marcelli, A., Wilkins, S., Ming, H., Tian, Y., Nugent, K., Zhu, P. & Wu, Z. Y. (2008). *Phys. Rev. A*, **78**, 023817.

[bb28] Maser, J., Osanna, A., Wang, Y., Jacobsen, C., Kirz, J., Spector, S., Winn, B. & Tennant, D. (2000). *J. Microsc.* **197**, 68–79. 10.1046/j.1365-2818.2000.00630.x 10620150

[bb29] McEwen, B., Downing, K. & Glaeser, R. (1995). *Ultramicroscopy*, **60**, 357–373. 10.1016/0304-3991(95)00082-8 8525549

[bb30] Nave, C. (2018). *J. Synchrotron Rad.* **25**, 1490–1504. 10.1107/S1600577518009566 PMC6140389 30179189

[bb31] Nave, C. (2019). *J. Synchrotron Rad.* **26**, 603–604. 10.1107/S1600577519002601 PMC6412174 30855273

[bb32] Robinson, I. (2015). *IUCrJ*, **2**, 477–478. 10.1107/S2052252515015109 PMC4547814 26306188

[bb33] Rodriguez, J. A., Xu, R., Chen, C.-C., Huang, Z., Jiang, H., Chen, A. L., Raines, K. S., Pryor Jr, A., Nam, D., Wiegart, L., Song, C., Madsen, A., Chushkin, Y., Zontone, F., Bradley, P. J. & Miao, J. (2015). *IUCrJ*, **2**, 575–583. 10.1107/S205225251501235X PMC4547825 26306199

[bb34] Rose, A. (1973). *Vision: Human and Electronic*. New York: Plenum Press.

[bb35] Sayre, D. (1952). *Acta Cryst.* **5**, 60–65.

[bb36] Schneider, G. (1998). *Ultramicroscopy*, **75**, 85–104. 10.1016/s0304-3991(98)00054-0 9836467

[bb37] Schropp, A. & Schroer, C. (2010). *New J. Phys.* **12**, 035016.

[bb38] Shahmoradian, S., Tsai, E. H. R., Diaz, A., Guizar-Sicairos, M., Raabe, J., Spycher, L., Britschgi, M., Ruf, A., Stahlberg, H. & Holler, M. (2017). *Sci. Rep.* **7**, 6291. 10.1038/s41598-017-05587-4 PMC5524705 28740127

[bb39] Shen, Q., Bazarov, I. & Thibault, P. (2004). *J. Synchrotron Rad.* **11**, 432–438. 10.1107/S0909049504016772 15310961

[bb41] Starodub, D., Rez, P., Hembree, G., Howells, M., Shapiro, D., Chapman, H. N., Fromme, P., Schmidt, K., Weierstall, U., Doak, R. B. & Spence, J. C. H. (2008). *J. Synchrotron Rad.* **15**, 62–73. 10.1107/S0909049507048893 18097080

[bb42] Stockmar, M., Cloetens, P., Zanette, I., Enders, B., Dierolf, M., Pfeiffer, F. & Thibault, P. (2013). *Sci. Rep.* **3**, 1927. 10.1038/srep01927 PMC3668322 23722622

[bb43] Tsai, E., Usov, I., Diaz, A., Menzel, A. & Guizar-Sicairos, M. (2016). *Opt. Express*, **24**, 29089. 10.1364/OE.24.029089 27958573

[bb44] Villanueva-Perez, P., Bajt, S. & Chapman, H. N. (2018). *Optica*, **5**, 450–457.

[bb45] Villanueva-Perez, P., Pedrini, B., Mokso, R., Guizar-Sicairos, M., Arcadu, F. & Stampanoni, M. (2016). *Opt. Express*, **24**, 3189–3201. 10.1364/OE.24.003189 26906983

[bb46] Xu, C., Hayworth, K., Lu, Z., Grob, P., Hassan, A., García-Cerdán, J., Niyogi, K., Nogales, E., Weinberg, R. & Hess, H. (2017). *eLife*, **6**, e25916. 10.7554/eLife.25916 PMC5476429 28500755

